# Visual motion information modulates tactile roughness perception

**DOI:** 10.1038/s41598-020-70831-3

**Published:** 2020-08-18

**Authors:** Yosuke Suzuishi, Souta Hidaka, Scinob Kuroki

**Affiliations:** 1 Department of Psychology, Rikkyo University, 1-2-26, Kitano, Niiza-shi, Saitama 352-8558 Japan; 2 NTT Communication Science Laboratories, Nippon Telegraph and Telephone Corporation, 3-1, Morinosato-Wakamiya, Atsugi, Kanagawa 243-0198 Japan

**Keywords:** Human behaviour, Somatosensory system

## Abstract

We perceive the roughness of an object through our eyes and hands. Many crossmodal studies have reported that there is no clear visuo-tactile interaction in roughness perception using static visual cues. One exception is that the visual observation of task-irrelevant hand movements, not the texture of task-relevant objects, can enhance the performance of tactile roughness discrimination. Our study investigated whether task-irrelevant visual motion without either object roughness or bodily cues can influence tactile roughness perception. Participants were asked to touch abrasive papers while moving their hand laterally and viewing moving or static sine wave gratings without being able to see their hand, and to estimate the roughness magnitude of the tactile stimuli. Moving gratings with a low spatial frequency induced smoother roughness perceptions than static visual stimuli when the visual grating moved in the direction opposite the hand movements. The effects of visual motion did not appear when the visual stimuli had a high spatial frequency or when the participants touched the tactile stimuli passively. These results indicate that simple task-irrelevant visual movement without object roughness or bodily cues can modulate tactile roughness perception with active body movements in a spatial-frequency-selective manner.

## Introduction

In daily life, when we touch an object with our hands, we automatically feel its texture. Texture information from the tactile modality consists of roughness, stickiness, slipperiness, and friction^1^. Specifically, roughness is one of the fundamental properties of texture information^2^. While we can perceive roughness through other sensory modalities besides touch (vision and audition), dominance of the tactile modality in roughness perception has been reported, especially for fine textures^3,4^.

Studies of crossmodal interactions have consistently demonstrated that our percepts consist of multisensory information^5,6^ that establishes robust and coherent percepts of the outer world^7^. In this context, modulatory effects of auditory information on tactile roughness perception^8–10^ have been reported that can be explained based on consistency in the temporal frequency domain^11^. In contrast to these audio-tactile interactions, visuo-tactile interactions in roughness perception have not been clearly reported^12^. It has been reported that the presentation of visuo-tactile information does not result in better performance in roughness discrimination than does the presentation of unimodal information for abrasive papers with the same roughness for vision and touch^13^. When different roughness information was presented to the visual and tactile modalities, the tactile modality appeared to be dominant over the visual modality^12,14,15^: Visuo-tactile matching of abrasive papers was more weighed on the tactile than the visual modality^16^ and visual roughness discrimination of fabric stimuli was biased by tactile stimuli, but the reverse was not observed^17^. These findings suggest that tactile roughness information is dominant over visual information, so that visual information has no effect on tactile roughness perception.

These absences of visual modulation of tactile roughness perception has been demonstrated by presenting static visual information (i.e., visual information without any movement) and dynamic tactile information (i.e., observers explored the tactile stimuli with their hands, or their hands were scanned by moving stimuli). However, when dynamic visual information is presented with dynamic tactile information, clear visuo-tactile interactions have been reported. Heller^18^ asked participants to explore a set of three abrasive papers by picking up, holding, and moving each stimulus freely. The participants were also asked to wear a cotton glove whose index finger was removed for tactile observations. Smoothness discrimination was performed unimodally (vision or touch) or crossmodally (vision and touch). Discrimination performance in the crossmodal condition was superior to that in the unimodal condition. Intriguingly, better performance in the crossmodal condition was still observed when the participants observed the abrasive papers through a half-transparent plastic plate so that visual roughness information was not available and only their hand movements were visible. Similar findings were reported in a classification task for textured objects^19^. These findings suggest that the presentation of dynamic visual cues, not only through the texture itself but also through the body (hand movements), can affect tactile roughness perception.

This type of visuo-tactile interaction, an effect of task-irrelevant visual information (i.e., body information) on a tactile task, has been demonstrated for tasks other than roughness perception^20–24^. The effects of dynamic visual information regarding the hands on tactile roughness perception^18,19^, as mentioned above, can be explained in the context of noninformative vision. We note that dynamic information on touch (i.e., spatiotemporal information on the body surface) itself plays an important role in roughness perception^25^, and visual motion information co-occurs with tactile dynamic information when we observe an object’s roughness crossmodally. Given that the presentation of dynamic visual information per se is also a key factor in visuo-tactile interactions in roughness perception due to the congruency of crossmodal dynamic information, visual motion without bodily cues or roughness information may also affect tactile roughness perception. To the best of our knowledge, no study has investigated the effects of simple, task-irrelevant visual motion information on tactile roughness perception. The current study examined whether and how visual motion information without bodily cues or roughness information affects tactile roughness perception.

In our experiments, we asked the participants to touch abrasive papers by laterally moving their right hand consistently with a visual reference maker. During the presentation of the tactile stimuli, a dynamic visual grating with a low spatial frequency was presented on a display covering the tactile stimuli and hiding the participant’s hand. The visual motion was presented in a spatially different location (the upper half of the display) from the tactile stimuli (beneath the lower half of the display). The speed of the visual motion was the same as the visual reference marker for hand movement, and the direction of the visual motion was either the same as (congruent condition) or opposite (incongruent condition) the hand movement. We also included a condition with a static visual grating as a baseline. After the presentation of these stimuli, the participants performed a magnitude estimation of the roughness of the abrasive paper. Experiment 1 showed that the presentation of visual motion incongruent with the hand movement induced a smoother roughness estimation than the baseline condition. Experiment 2 replicated these findings, and further revealed that the velocity of participants’ recorded hand movements and the perceived velocity of the visual reference marker for hand movements did not correlate with the roughness estimation in any visual conditions among participants, indicating that the visual effect observed in the incongruent condition was not due to the modulation of hand movements and/or visual motion contrast between the grating and reference marker. Experiments 3 and 4 respectively demonstrated that the effects of visual motion did not appear when the moving visual stimuli had a high spatial frequency or when the participants touched tactile stimuli passively. These results suggest that simple visual motion without information on the hand and roughness, whose direction is opposite the hand movements, can modulate tactile roughness perception with active body movements in a spatial-frequency-selective manner.

## Methods

### Participants

Sixty people (15 males and 45 females; 18–48 years old) participated in the experiments. Each experiment involved 15 participants. All of them reported normal or corrected normal vision and normal touch. They were naive to the purpose of the experiment.

### Apparatus and stimuli

Tactile roughness perception differs between finer and coarser textures^25^. Dynamic touch is assumed to mainly contribute to the percept of roughness, specifically for finer textures^25^. Since the current study asked the participants to touch the stimuli dynamically, we presented relatively finer abrasive papers (P180, P400, P600, P800, and P1000 in meshes following the Japanese Industrial Standards; the average particle diameters were 75, 35, 25.8, 21.8, and 18.3 μm, respectively) as the tactile stimuli. The size of the abrasive papers was 23 × 28 cm in Experiments 1–3, while in Experiment 4, the size of the abrasive papers was 6 × 22.5 cm. The abrasive papers were set in a cardboard frame on a desk to fix their position while they were being touched in Experiments 1–3. In Experiment 4, we set the abrasive papers on a motorized x-stage (CKD, ERL2-45E06-50BM-R3F2). The abrasive papers moved leftward at 13.5 cm/s for 1.67 s. White noise bursts were presented through headphones (Sennheiser, HDA 200) in Experiment 4 in order to prevent the participant from hearing artificial noises from the x-stage.

**Figure 1:**
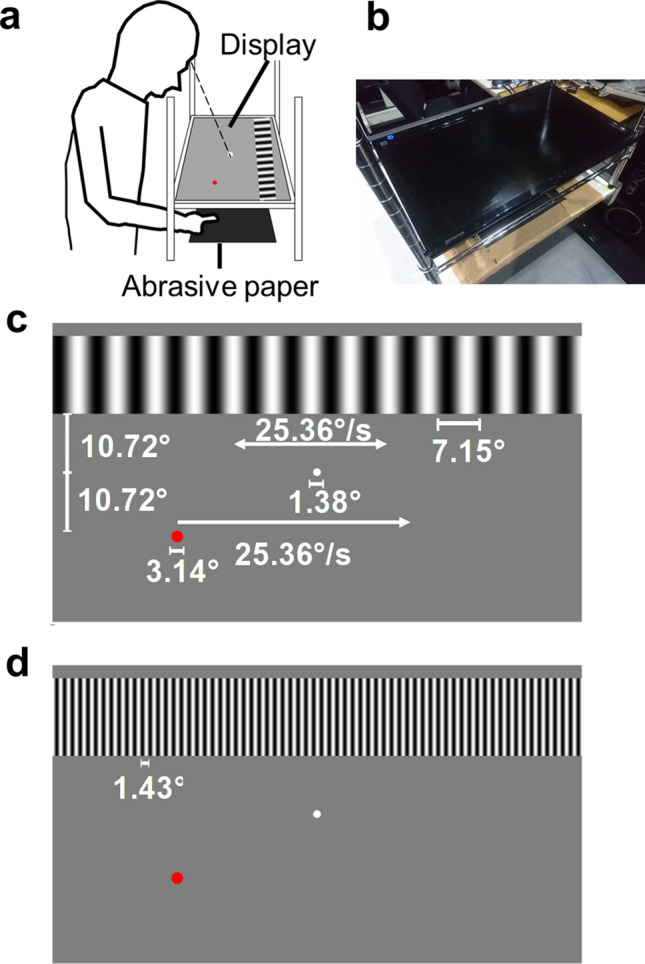
Schematic illustrations of the experimental setups and stimuli. (**a**) Tactile stimuli (abrasive paper) and participants’ hands were placed on a table. A visual display sat on a hand-made aluminum stand above them. Participants were asked to touch the abrasive paper with their index finger of the right hand moving rightward. (**b**) Photograph of the experimental setups in Experiments 1, 2, and 3. (**c**) Visual stimuli used in Experiments 1 and 2. The visual grating stimuli had a low spatial frequency (0.14 cycles per degree). A white marker was presented at the center of the display as a fixation point. A moving red marker was presented as a reference for the participants’ hand movements. In Experiment 4, we presented the same visual stimuli except that the red reference marker for hand movements was not presented. (**d**) Visual stimuli used in Experiment 3. The grating had a higher spatial frequency (0.70 cycles per degree) than in the other experiments.

Visual stimuli were presented on a linearized LCD (LG, D2342, 1,360 × 768 pixels, 60 Hz) using a customized computer (Dell, Precision T3500) and MATLAB (MathWorks, Inc.) with the Psychophysics Toolbox^26,27^ for Experiments 1–3. In Experiment 4, the visual stimuli were presented on a linearized LCD (DELL, U2311Hb, 1920 × 1,080 pixels, 60 Hz) using a computer (Dell, Vostro 3,268) and Python with Psychopy^28,29^. In all experiments, the display was set laterally on a hand-made aluminum stand above the tactile stimuli so that the participants could see neither their hand nor the tactile stimulus during the experiments (Fig. 1a,b). The viewing distance was 30 cm. The background of the display was set to gray (44.00 cd/m^2^). On the display, a sine wave grating (0.15–88.20 cd/m^2^: mean = 44.00 cd/m^2^) was presented as static or moving at 14.25° in height and 80.73° in width (corresponding to the width of the display) (Fig. 1c,d). We presented the sine grating because we could show visual motion with a minimum modulation of one stimulus dimension (luminance)^30^ and without roughness or bodily cues. The moving grating shifted rightward or leftward at 25.36°/s. The absence of clear visuo-tactile interactions in tactile roughness perception has been reported for relatively finer visual roughness^14^. This may be due to a low discriminant ability for finer roughness in vision^3^. Furthermore, vision studies have reported that relatively coarser visual roughness is discriminable and utilized for visual roughness estimation^15,31^. Based on these findings, we presented a sine wave grating with a relatively low spatial frequency (7.15° in wavelength, 0.14 cycles per degree) for Experiments 1, 2, and 4 (Fig. 1c). In Experiment 3, we presented a visual grating that was 1.43° in wavelength (0.70 cycles per degree), a spatial frequency five times higher than in the other experiments (Fig. 1d). We also presented a white circle (0.69° in radius) at the center of the display as a fixation point and a red marker (1.07° in radius) moving rightward from the left side of the display at 25.36°/s for 1.67 s as the reference for hand movements. The distance between the fixation point and the reference maker and between the fixation point and the grating was 10.72°. In Experiment 4, we did not present the reference marker because the participants touched tactile stimuli passively without moving their hands. The duration of the presentation of the tactile and visual stimuli was 1.67 s.

In Experiment 2, we also used a video camera (SONY HDR-SR12, with a 30 Hz refresh rate) to record the participants’ hand movements. The video camera was set in front of the table. A flashlight was also set on the table to illuminate the participants’ hands. A ruler was also attached to the table to record the hand movement distance and to calculate hand movement velocity.

### Procedure

We performed four experiments. Experiment 1 presented the visual stimuli with a low spatial frequency (0.14 cycles per degree, Fig. 1c) and asked the participants to touch the tactile stimuli with active hand movements. We conducted Experiment 2 using the same procedure as Experiment 1 to replicate the findings. Furthermore, we recorded the participants’ hand movements during the presentation of the comparison stimuli and measured the perceived velocity of the reference marker. In Experiment 3, the stimuli and procedures were identical to those in Experiment 1, except that visual gratings with high spatial frequency (0.70 cycles per degree) were presented (Fig. 1d). The stimuli and procedures in Experiment 4 were also identical to those in Experiment 1, except that the participants were asked to touch the tactile stimuli passively.

For all experiments, we adopted a magnitude estimation method to measure the perceived magnitude of roughness. The participants sat on a chair in front of a table. They were asked to place their right hand underneath the display on the table (Fig. 1a,b). We used a tactile stimulus with intermediate physical roughness (i.e., P600-mesh abrasive paper) as the standard stimulus. One of the five abrasive papers, including a P600 mesh, was presented as the comparison stimulus. At the beginning of each trial, the standard stimulus was presented without the visual grating. Following a 10-s interval after the presentation of the standard stimulus, the comparison stimulus was presented together with the visual grating (Fig. 1c,d). After the presentation of the comparison stimulus, the participants orally reported the subjective roughness of the comparison stimulus on a 10-point scale (where 1 corresponded to the smoothest and 10 to the roughest) compared to the standard stimulus, whose perceived roughness value was set at 5. Further, the participants reported the perceived direction of the visual motion (left or right) or the absence of movement. The fixation point was presented on the display, and the participants were asked to fixate on it during the trial. In Experiments 1–3, the reference marker for hand movements was also presented. The presentation of the tactile stimuli was terminated when the visual stimuli disappeared.

In Experiments 1–3, the participants were asked to place their right index finger on the leftmost side of the tactile stimulus and move their finger from left to right on the tactile stimulus to accompany the visual reference marker’s movement (active scan). In Experiment 4, the participants were asked to place their right or left index finger on the tactile stimulus and keep it still while the tactile stimulus moved leftward (passive scan).

We introduced three visual movement conditions: congruent (rightward visual motion with rightward hand movement), incongruent (leftward visual motion with rightward hand movement), and baseline (static visual stimuli with rightward hand movement). In Experiment 4, the leftward and rightward visual motions against leftward motion by the tactile stimuli (i.e., the spatiotemporal properties of touch corresponding to rightward hand movement) were defined as congruent and incongruent, respectively. Each participant performed 45 trials in all (3 visual movement conditions × 5 tactile comparison stimuli × 3 repetitions) in Experiments 1–3, while Experiment 4 involved 120 trials in total (3 visual movement conditions × 5 tactile comparison stimuli × 8 repetitions). The presentation orders of the visual movement conditions and tactile comparison stimuli were randomized for each participant and counterbalanced across them.

Before the main experiment, each participant completed a practice session. In Experiments 1–3 (active scan condition), the participants moved their hand over the standard stimulus three times. In Experiment 4 (passive scan condition), the participants conducted five trials with five different roughness stimuli in random order.

Experiment 2 recorded the participants’ hand movements during the presentation of the comparison stimuli for 1.7 s in each trial. An additional session was also introduced to measure the perceived velocity of the reference marker. The reference marker was first presented without the visual grating and subsequently presented with one of the grating stimuli in the same manner as in the tactile roughness estimation session (Fig. 1c). After the presentation of the visual stimuli, the participants were asked to orally report the perceived velocity of the reference marker in the second presentation (comparison) compared to that in the first presentation (standard, set as 5 on the scale) on a 10-point scale (where 1 corresponded to the slowest and 10 to the fastest). This session was performed for nine trials (three visual movement conditions × three repetitions).

### Data analysis

We analyzed all individual data for the perceived magnitude of roughness using the generalized linear mixed model (GLMM) with a Gaussian distribution. Since we focused on the effect of visual motion on the perceived roughness, the visual movement condition was set as a fixed effect (slopes), and the particle diameters of the abrasive papers and the participants’ responses were set as a random effect (intercepts). We performed a one-way full-factorial analysis of variance (ANOVA) for fixed effects^32^. We further performed multiple comparisons with a corrected alpha level (*p* < 0.05) using the Holm-Bonferroni method to compare the fixed effect across visual movement conditions when the ANOVA showed a significant main effect.

In Experiment 2, we calculated the hand movement velocity in each condition for the trials with the P600-mesh comparison stimulus having the same roughness as the preceding standard stimulus. We divided the data into 17 time bins (100 ms interval in each time bin) because the obtained hand movement velocities were not linear. We then performed a two-way repeated-measures ANOVA (3 visual movement conditions × 17 time bins). We also analyzed the perceived velocity of the visual reference marker with GLMM. The visual movement condition was set as a fixed effect (slopes), and the participants’ responses as a random effect (intercepts). We then performed an ANOVA for the fixed effects. We further performed correlation analyses between the perceived roughness and the hand movement velocity or the perceived velocity of the visual reference marker in each visual movement condition. For the calculations of correlation coefficients, we averaged the perceived magnitudes of roughness across all comparison stimuli and trials in each visual movement condition. We also calculated the mean hand movement velocities across all time bins and the mean perceived velocity of the visual reference marker across the trials in each visual movement condition.

Analyses with GLMM were performed using R^33^ and RStudio^34^ with lme4^35^ and lmerTest^36^. Other statistical tests were conducted using JASP^37^.

## Results

### The judgments of visual stimuli

The proportions of correct responses for the judgments of the visual motion direction or absence of visual motion were above 0.98 (chance level = 0.33) in all experiments, showing that the participants perceived the visual motion correctly.

### Effects of visual motion on tactile roughness perception

#### Experiment 1

**Figure 2:**
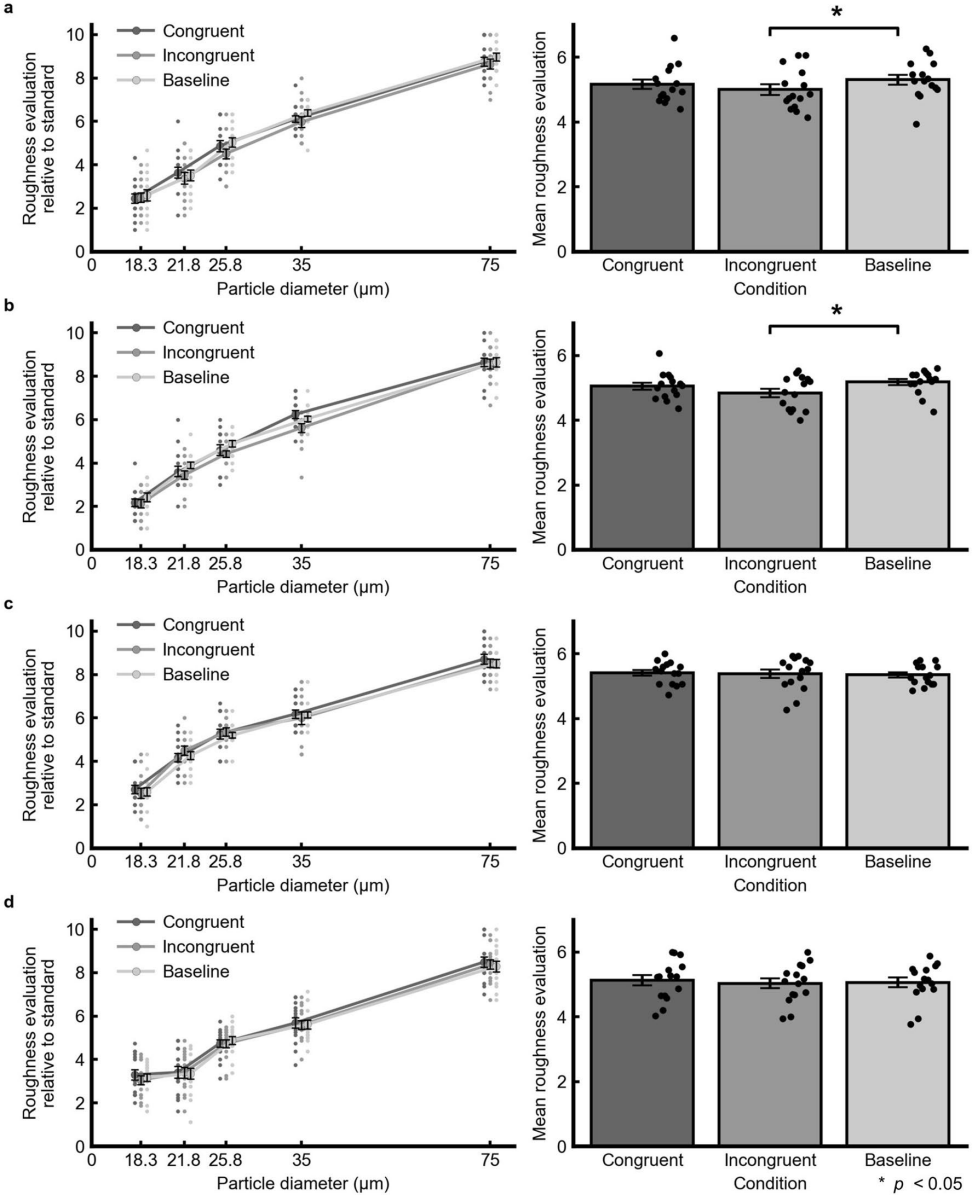
Results for tactile roughness judgments in Experiments 1–4 (a–d). The left-hand panels show the mean roughness evaluation of each comparison stimulus relative to the standard stimulus. The horizontal axis denotes the particle diameter, such that larger values indicate higher physical roughness. The right-hand panels show the perceived roughness magnitudes averaged across the comparison stimuli. The small dots represent each participant’s data. The error bars denote the standard errors of the mean (*N* = 15). Asterisks indicate significant differences in the fixed effect with GLMM (*p* < 0.05).

In Experiment 1, we presented a visual grating with a low spatial frequency (0.14 cycles per degree; Fig. 1c) in order to investigate the effect of visual motion without bodily cues or roughness information on tactile roughness perception. We asked the participants to evaluate the perceived roughness for each tactile comparison stimulus in comparison to the standard stimulus (Fig. 2a, left). The perceived roughness magnitudes averaged across the comparison stimuli in each visual movement condition were as follows: congruent: 5.16; incongruent: 5.00; baseline: 5.31 (Fig. 2a, right). Full-factorial ANOVA with the GLMM model showed a significant difference for the fixed effects of the visual movement conditions [*F*(2, 654) = 4.49, *p* = 0.012]. Multiple comparisons showed that the perceived roughness in the incongruent condition was smaller than in the baseline condition [M =  − 0.31, *t*(654) = 3.00, *p* = 0.003]. The differences were not significant between the congruent and baseline conditions [M  =  − 0.14, *t*(654) = 1.39, *p* = 0.17] or between the congruent and incongruent conditions [M = 0.16, *t*(654) = 1.61, *p* = 0.11].

#### Experiment 2

The aim of Experiment 2 was to replicate the findings of Experiment 1. We thus repeated the same procedures as Experiment 1 for the other participants. The mean perceived roughness magnitudes were as follows: congruent: 5.05; incongruent: 4.84; baseline: 5.18 (Fig. 2b). Full-factorial ANOVA showed a significant difference for the fixed effects of the visual movement conditions [*F*(2, 653.97) = 5.84, *p* = 0.003]. Multiple comparisons revealed a significant difference between the incongruent and baseline conditions [M =  − 0.34, *t*(654.1) = 3.38, *p* < 0.001], but not between the congruent and baseline conditions [M =  − 0.13, *t*(654.1) = 1.26, *p* = 0.21] or congruent and incongruent conditions [M = 0.21, *t*(654) = 2.12, *p* = 0.035]. These results clearly replicate the findings of Experiment 1.

**Figure 3:**
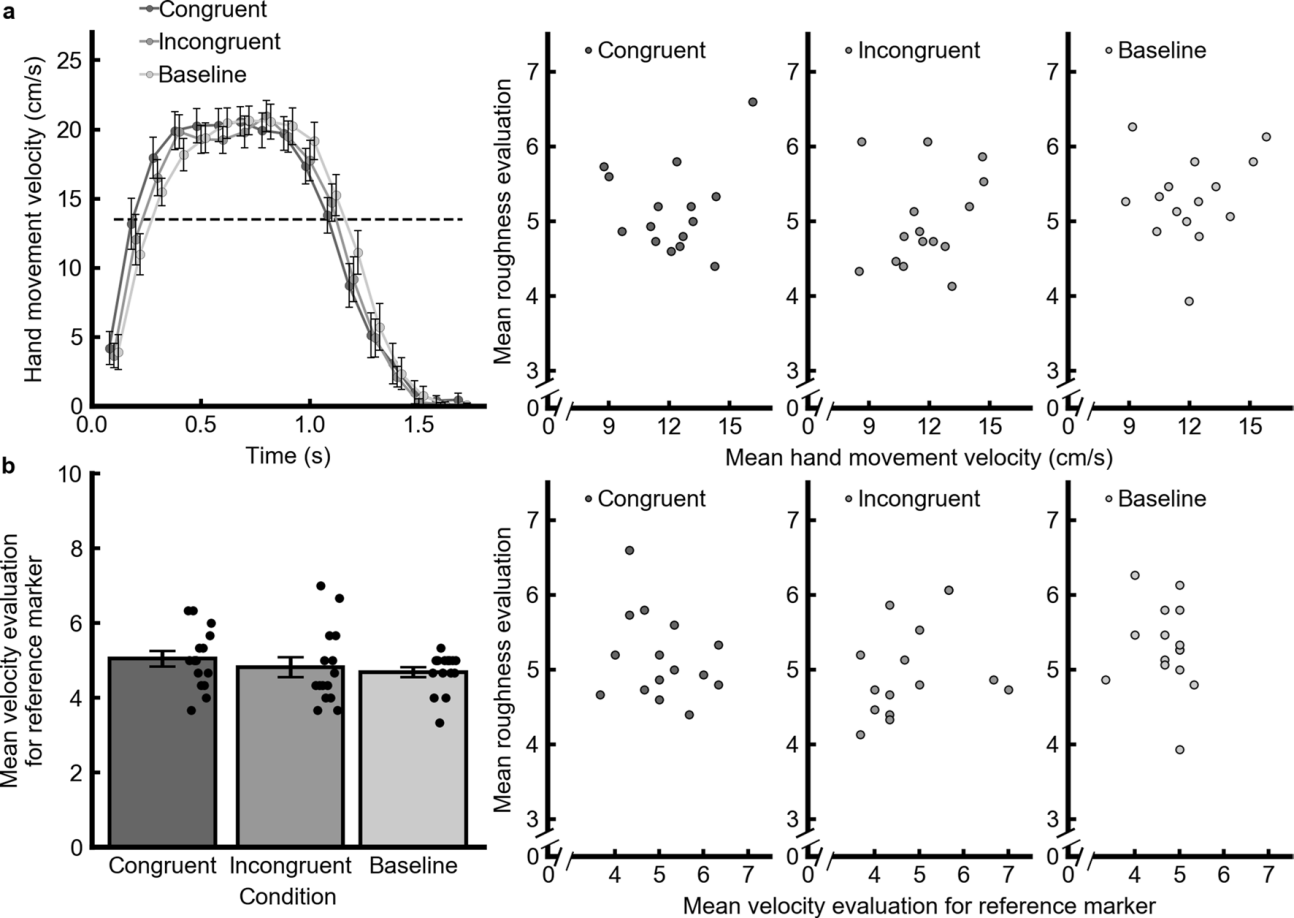
(**a**) Results for hand movement velocity in Experiment 2. The leftmost panel shows the mean hand movement velocities for each condition plotted for each time bin (0.1 s). The dashed line denotes the physical velocity of the visual reference marker. Right-hand panels show scatter plots of the perceived roughness magnitude against the hand movement velocity in each visual movement condition. (**b**) Results for the perceived velocity of the visual reference marker in Experiment 2. The leftmost panel shows the mean perceived velocity of the reference marker in each condition. The small dots represent each participant’s data. Right-hand panels show scatter plots of the perceived roughness magnitude against the perceived velocity of the visual reference marker in each condition. The error bars denote the standard errors of the means (*N* = 15).

It has been reported that changes in hand movements induce differences in spatiotemporal information on the skin surface and affect tactile roughness perception^38^. We therefore analyzed the participants’ hand movement velocities when the participants hepatically scanned a comparison stimulus identical to the standard one. We divided the nonlinear hand movement velocity data into 17 time bins (100 ms interval in each time bin) (Fig. 3a, left-most). The two-way repeated-measures ANOVA with the factors of visual movement conditions (congruent, incongruent, and baseline) and time bins revealed a significant main effect of the time bins [*F*(16,224) = 65.667, *p* < 0.001, *η*^*2*^ = 0.75]. The main effects of the visual movement conditions [*F*(2, 28) = 1.45, *p* = 0.25, *η*^*2*^ = 0.000] and the interaction [*F*(16,192) = 1.32, *p* = 0.12, *η*^*2*^ = 0.004] were not significant. We also investigated whether the perceived velocities of the reference marker differed across the visual conditions. The mean perceived velocity of the reference marker in each visual movement condition was as follows: congruent: 5.04; incongruent: 4.82; baseline: 4.69 (Fig. 3b, left-most). The full-factorial ANOVA of the fixed effects of visual movement condition found no significant main effect [*F*(2,0.08) = 1.49, *p* = 0.23].

We also calculated correlation coefficients between the perceived roughness magnitude in each visual movement condition and the hand movement velocities averaged across the time bins or the perceived velocity of the visual reference marker. We found no significant correlations for either hand movement velocities (congruent: *r* = 0.13, *p* = 0.64; incongruent: *r* = 0.21, *p* = 0.46; baseline: *r* = 0.15, *p* = 0.60) (Fig. 3a, right) or the perceived velocity of the visual reference marker (congruent: *r* =  − 0.26, *p* = 0.35; incongruent: *r* = 0.31, *p* = 0.26; baseline: *r* =  − 0.15, *p* = 0.60) (Fig. 3b, right).

#### Experiment 3

This experiment investigated whether the visual effect on tactile perceived roughness observed in Experiment 1 also appeared for a different spatial frequency of the visual gratings. We presented a visual grating whose spatial frequency was five times higher (0.70 cycles per degree) than in Experiments 1 and 2 (Fig. 1d). The mean perceived roughness magnitudes were as follows: congruent: 5.41; incongruent: 5.38; baseline: 5.35 (Fig. 2c). The full-factorial ANOVA did not show a significant difference for the fixed effects of the visual movement conditions [*F*(2, 654) = 0.18, *p* = 0.84].

#### Experiment 4

Experiment 4 tested whether the visual effect on tactile perceived roughness observed in Experiment 1 also appeared with passive touch. The mean perceived roughness magnitudes were as follows: congruent: 5.12; incongruent: 5.03; baseline: 5.06 (Fig. 2d). The full-factorial ANOVA did not find a significant difference for the fixed effects of the visual movement conditions [*F*(2, 1779) = 1.07, *p* = 0.34].

## Discussion

Studies of visuo-tactile interactions with roughness perception have consistently shown that static visual roughness information cannot affect tactile roughness perception^12^. However, it has been reported that seeing hand movements without object roughness information modulated the performance of tactile roughness discrimination^18^. The current study investigated whether simple visual motion (i.e., sine wave gratings) without bodily cues or object roughness information affected tactile roughness perceptions of abrasive paper. Our findings provide the first demonstration that simple, task-irrelevant visual movement modulates tactile roughness perception. We found that visual motion with low spatial frequency and horizontal direction opposite that of the active hand movement (the incongruent condition) induced smoother tactile roughness perception than the situation where the static visual stimulus was presented (the baseline condition) (Experiments 1 and 2). This visuo-tactile interaction was not observed when the visual motion had a high spatial frequency (Experiment 3) or when the participants touched the stimuli passively (Experiment 4).

Thus far, visuo-tactile interactions in motion perception have been demonstrated. The movement of visual gratings altered the perceived speed of the tactile grating movement^39^, and the visual motion of dot patterns affected motion direction judgments of tactile dot patterns even when the visual and tactile motion were presented in different positions^40^. The motion aftereffects were reported as transferring between vision and touch^41^. We should note that in our experiments, the participants were asked to report not only the perceived roughness of the abrasive paper but also the direction or absence of visual grating movement, so they needed to pay attention to the visual motion. Nonetheless, the effects of visual motion did not appear when the visual motion moved in the same direction as the hand movement or when the participants touched the abrasive paper passively. We could assume that the simple visuo-tactile interactions in motion perception cannot explain our findings.

One may argue that the effect of simple visual motion on tactile roughness perception is related to tactile memory^42^. In our experiments, the temporal interval between the standard and comparison stimuli was 10 s. During this interval, the memory of the standard stimulus decayed, and consequently the participants’ roughness judgments might shift toward a smoother value. We note that the presentations of the standard and comparison stimuli were identical in all experiments. If tactile memory affects tactile roughness perception, the effects should be observed equally in all conditions across all experiments. However, we found differences in tactile roughness perception between the incongruent and baseline conditions only in Experiments 1 and 2. These results cannot be explained by the effects of tactile memory. The involvement of attentional effects such as distractions from tactile stimuli and/or inhibition of return^43^ from visual to tactile stimuli may also be assumed. The presentation of the incongruent visual motion may induce an attentional capture and/or attentional distraction toward the visual motion and reduce or divert attention to tactile stimuli, whereupon the perceived roughness becomes smoother. It should be noted that the modulatory effects of visual motion on perceived tactile roughness were not observed in Experiment 3, where the spatial frequency of the visual stimuli was higher, or Experiment 4, where the tactile stimuli were presented passively, although the incongruent visual motion was presented. We can thus assume that the effects of visual motion on tactile roughness perception are not simply explained by attentional capture and/or attentional distraction.

One plausible explanation for the effect of visual motion incongruent with hand movement on tactile roughness perception is the perceptual expectation of friction-like sensation on actual tactile inputs induced by the incongruence in motion direction between visual motion and hand movements. It has been demonstrated that the temperature of a blue object is felt to be warmer than a red object or an object touched with the eyes closed^44^. When we see a blue object, we spontaneously expect its temperature to be cooler, so our brain perceives the object to be warmer than it is when the perceived temperature of a blue object is not as cold as expected. It has also been shown that the presentation of visual motion incongruent with exploring hand movements (where the visual motion is slower than the hand movements) induces a pseudo-haptic friction sensation on the touched surface^45^. In our experiment, the participants moved their hand laterally while seeing horizontal visual motion opposite the hand movements. This incongruence might induce the exaptation/interpretation that the hand movements were not smooth because the touched surface was coarser, then because the touched surface had the same particle diameters across visual movement conditions, tactile roughness was perceived as smoother by this expectation of friction-like sensation in the incongruent condition versus the baseline condition. Such a perceptual expectation may not occur in the congruent condition because there was no conflict in motion direction between visual motion and hand movements.

Another possible explanation is that hand movements were modulated by visual motion information. Changes in hand movement induce modulations of spatiotemporal information on the skin surface, which would contribute to tactile roughness perception^38^. In fact, a minor effect of the mean velocity of hand movement on tactile roughness perception has been reported^46^. Another previous study^47^ also reported that tactile detection performance was at chance level when the exploring speed of hand exceeded 20 cm/s. In the current study, although the averaged hand movement velocities were below 20 cm/s, the peak velocity of hand movements seemed to be stable at approximately 20 cm/s (Fig. 3a). This suggests that hand movement velocity affects tactile roughness perception. However, we did not find any differences in hand movements between the visual movement conditions and no significant correlations between the perceived roughness magnitudes and the hand movement velocity in any visual movement condition (Fig. 3a). It thus appears that the changes in hand movement velocity did not play a critical role in the effects of visual motion on perceived tactile roughness, at least under our experimental conditions. It may also be possible that the perceived velocity of the reference marker for hand movements was modulated by the incongruent visual motion, for example, based on the visual motion contrast effect. However, we found no differences in the perceived velocity of the reference marker across conditions and no significant correlation between the perceived roughness magnitudes and the perceived velocity of the reference marker in any visual movement condition (Fig. 3b). The changes in the perceived velocity of the reference marker thus cannot explain the effect of visual motion on tactile roughness perception.

Consistent with previous findings regarding visuo-tactile roughness perception^18,19^, our results showed that the visual effects on tactile roughness perception were observed only with active touch (Experiments 1 and 2). The effects of simple visual motion on tactile roughness perception did not appear when the tactile stimulation was presented passively (Experiment 4). The inconsistency of the results between Experiments 1 and 2 and Experiment 4 can only be explained by the difference in the manner of touch (active vs. passive) because the stimuli and procedures were almost identical apart from the manner of touch across the experiments. Tactile surface discrimination performance was reported to be enhanced during active exploration relative to passive touch^18^, whereas tactile vibratory discrimination performance was degraded during active tactile exploration compared to a situation of no hand movement^49^. We might argue that active hand movements are associated with this tactile perceptual process, gaining information about the surface properties, so that our visuo-tactile interaction occurred only with active hand movements.

Our findings suggest that simple visual dynamic information and active body movements are key factors in inducing visuo-tactile interactions in roughness perception, in line with previous findings^18,19^. Some fMRI studies have reported shared brain activity for visual and tactile texture perception. The visual image of a body part being touched induced activation in the secondary somatosensory cortex (S2)^50^. Similarly, the observation of another person’s body being touched induced activation in the primary somatosensory cortices (S1) as well as S2^51^. With respect to tactile roughness perception, stronger posterior S1 activations were observed when the participants saw dynamic visual images touching their own or others' bodies during a tactile roughness discrimination task than when only the visual images were presented^52^. S1 was also reported to be activated by visual texture images related to tactile sensation (roughness), but not by those unrelated to tactile sensation (color)^53^. Furthermore, shared activations were reported for visual and tactile texture perception in a higher cortical region (the medial occipital cortex)^54,55^. These shared cortical processes in both the lower somatosensory and higher cortical regions might be involved in the visual modulations of tactile roughness perception observed here.

The effect of the incongruent visual motion on tactile roughness perception was observed only for the visual grating having a low spatial frequency (0.14 cycles per degree, Fig. 1c) and not for a grating with higher spatial frequency (0.70 cycles per degree, Fig. 1d) (Experiment 3). It has been demonstrated that motion detection sensitivity was greater for higher spatial frequency gratings than for lower spatial frequency gratings at a relatively low velocity^48^. This implies that the visual motion of the gratings with a high spatial frequency was well perceived in our experimental situation with the relatively low velocity of the visual gratings, and the differences in sensitivity to visual spatial frequency cannot explain the effect of visual motion on tactile roughness perception. We speculate that the expectation of friction-like sensation induced by the incongruence in motion direction between visual motion and hand movements may predominantly occur with low-frequency visual gratings. Further studies should directly investigate this point.

We should also note that the current study measured hand movements just in a lateral, one-dimensional space with a low-frequency visual grating, even though people can haptically explore objects in any direction in daily life. Future studies need to conduct detailed investigations of hand movements in two- or three-dimensional space while presenting visual gratings of different spatial frequencies. Furthermore, investigations of the effects of simpler visual motion stimuli like visual cursor movements are worth performing to test possible effects of simple visual motion without spatial frequency information on tactile roughness perception. These detailed manipulations of the properties of visual motion and hand movement could improve our understanding of the mechanisms underlying visuo-tactile integrations in roughness perception.

### Ethics statement

The experimental procedures were approved by the local ethics committee of Rikkyo University and the NTT Communication Science Laboratory and were performed in accordance with the approved guidelines and the Declaration of Helsinki. Informed consent was obtained from each participant before each experiment.

## Data Availability

The datasets generated and/or analyzed in the current study are available from the corresponding author upon reasonable request.
